# Moral diversity and the challenge of responsibility in AI-CDSS

**DOI:** 10.1186/s13010-026-00226-1

**Published:** 2026-07-01

**Authors:** Wenke Liedtke, Martin Langanke

**Affiliations:** 1 Faculty of Theology, University of Greifswald, Greifswald, Germany; 2 Department of Social Work, Protestant University of Applied Sciences Rhineland-Westphalia-Lippe, Bochum, Germany

**Keywords:** Artificial intelligence, Ethical pluralism, Moral diversity, Clinical decision support systems, Responsibility, Machine learning, Patient-healthcare professional relationship

## Abstract

The increasing integration of artificial intelligence in clinical decision support systems (AI-CDSS) has fueled expectations of more personalized and effective diagnostics and therapies. By incorporating machine learning methods, AI-CDSS promise enhanced predictive accuracy, improved stratification, and innovative individualized care. However, this technological optimism is accompanied by complex ethical challenges, including issues of explainability, trust, autonomy, and data security. At the core of these debates lies the question of responsibility, which involves both its attribution and diffusion, as well as the underlying normative standards guiding moral action. In the context of healthcare practice, responsibility is further complicated by moral diversity—the coexistence of varying moral values, cultural beliefs, and ethical frameworks among healthcare professionals, patients, and institutional stakeholders. This plurality challenges the establishment of a unified normative standard necessary for ethically sound responsibility attribution. This paper offers an analysis of moral diversity and AI-CDSS as a challenge for responsibility in healthcare environments. Using a relational concept of responsibility the study examines key areas in which moral diversity affects responsibility in AI-mediated decision-making. This includes algorithmic bias, healthcare professional and patient interaction and the role of patients. Through these examples, the paper explains how different normative standards intensify ethical complexity in AI-supported clinical contexts. It argues that greater ethical sensitivity to moral diversity is essential—both in the development of AI-CDSS and in their application within morally value-laden healthcare situations.

## Introduction

Clinical decision support systems (CDSS) are widespread and well-established tools in healthcare. Against the backdrop of digitalization, these systems are gaining renewed importance as methods of Artificial Intelligence (AI). In particular, machine learning promises significant advances in predictive capabilities. Accordingly, AI-based clinical decision support systems (AI-CDSS) are increasingly applied and researched across various medical disciplines. Examples include radiology to detect tumors in imaging data [1], dermatology to classify skin lesions [2], as well as other fields such as cardiology or ophthalmology [3, 4]. These systems aim to improve time efficiency, streamline clinical processes, reduce workload, and enhance individualized outcomes [5–7].

The improvements are primarily based on the shift from rule-based to data-driven methods, which allows for greater dynamic and variety in distinguishing different cases. Through machine learning, systems can recognize, learn, and refine patterns and continuously compare and optimize performance [8, 9]. As a result, diagnoses and therapeutic recommendations can be better tailored to the individual patient.

From an ethical perspective, however, CDSS are associated with a range of well-documented challenges. Some challenges in traditional rule-based systems are well understood and manageable, while data-driven AI systems introduce new and complex ethical challenges [10]. These address fundamental aspects of medical ethics and healthcare practice, including explainability, autonomy, trust, privacy, and justice [11–15]. Among these, one of the most pressing challenges concerns the question of *responsibility*.

Responsibility, as a key principle for the ethical evaluation of technologies, plays a pivotal role in assessing the legitimacy and moral adequacy of AI-CDSS. Its debate can encompass legal and institutional questions of accountability and liability, as well as questions of remaining challenges and gaps in AI-use [16]. Moreover, it raises questions regarding diffusion of responsibility, about shifting professional roles and the development of expertise [17–19].

This paper therefore focuses on the challenge of a *normative standard* within the context of moral diversity and its relation to ethical responsibility in AI-CDSS. It examines the ethical implications and potential impacts of data-driven CDSS on physician–patient interactions through selected examples. Methodologically, a relational concept of responsibility is applied as an analytical framework to examine the *normative standard* as a central element of responsibility attribution. Against this background, the paper argues that existing approaches to responsibility in AI-CDSS fail to adequately acknowledge the existence of plural normative standards. Therefore, we propose relational responsibility as a framework that recognizes responsibility as a dynamic and context-sensitive relation that needs to address diverse moral perspectives.

Subsequently, selected challenges arising from moral diversity in the development and use of AI-CDSS are discussed and their implications for future approaches to moral diversity and responsibility in healthcare are examined.

## Responsibility and the normative standard

The concept of responsibility is central in ethical discourse on AI, which is mostly concerned with the attribution and evaluation of actions. It determines how actions are assessed, justified, and ascribed to individuals and collective agents. Therefore, it can play a crucial role in structuring decision-making processes in situations of uncertainty or vulnerability affecting healthcare professionals and patients.

In the literature, discussions range from questions of collective and individual responsibility [20, 21] and retrospective and prospective orientations [22], to thematic issues such as blame [23, 24], trust [25], and autonomy [26, 27]. Despite these diverse approaches and perspectives, all of them address the central question of how responsibility can be meaningfully attributed and distributed.

Responsibility may also be understood as a *relational* concept—an analytical framework that structures responsibility by focusing on certain relata [28]. It thereby enables the ethical assessment of complex systems such as data-driven CDSS. Here responsibility can be described as a structural, context-dependent configuration of relations [29]. These relations involve, for instance, agents, actions, and normative standards, which form the basic elements of responsibility attribution. In this sense relational responsibility does not primarily focus on interpersonal relations in a narrow sense (e.g. care ethics), but a broader framework of responsibility attribution and distribution, which is shaped by the interplay of multiple actors, institutional settings, and normative expectations and constraints [30]. This broader framework also includes system-level constellations, such as organizational structures and digital infrastructures, as well as technologically based constellations in the case of AI-CDSS.

This understanding differs from approaches that ground responsibility in a universally valid normative standard applicable across all contexts, such as Jonas, who extends responsibility to the long-term consequences of technological action [31]. Such rather deontological traditions focus mostly on universal duties and leave little room for adaption to a diverse moral landscape. This limitation becomes particularly relevant in medical and nursing contexts, where multiple and potentially conflicting normative standards can coexist in decision-making processes. At the same time, this does not imply that responsibility must be understood as exclusively relational or non-relational. Both approaches can coexist and respond to different normative and practical challenges in medical ethics and technology development.

Reflections on responsibility as a normative orientation can be found in the work of Jonas, within a more strongly duty-based framework of the ecological imperative [31]. Broader sociological and political perspectives, such as those of Weber, highlight the contextual nature of responsible action in practical decision-making [32]. In analytical terms, Feinberg distinguishes between causal and normative responsibility. He shows that responsibility attribution presupposes a normative framework of justification [33]. This means that responsibility cannot be assigned without prior evaluative criteria that determine what counts as justified action.

Building on this distinction, subsequent debates in the ethics of technology and medical ethics have further specified this. In contemporary debates, relational perspectives have been further developed. Van de Poel conceptualizes responsibility as a relational and distributed concept in complex socio-technical systems. He distinguishes between backward-looking and forward-looking dimensions of responsibility [34]. In contrast, Coeckelbergh emphasizes responsibility as answerability. This highlights the relational structure between agents and those affected–moral patients– which makes responsibility an almost ‘dialogical matter’ (p. 2061) [35].

Taking these various perspectives into account, responsibility cannot be stabilized through a single framework. Hence, relational responsibility here emphasizes that responsibility attribution is shaped by plural and contested normative standards. It assumes the existence of contextually varying moral perspectives that guide, for instance, physician–patient interaction and decision-making processes.

Within this framework, particular attention must therefore be given to the normative standard, which determines the moral and ethical basis of responsibility itself.

While other relata—such as the subject, the addressee, or the authority—are relevant for the detection and distribution of responsibility, the normative standard serves as the decisive criterion for whether and in what manner responsibility can be ascribed (see Table 1).

**Table 1:** Relational structure of responsibility: variables, definition, terms, and suitable insertions [29]

	Variable	Definition	Term	Suitable insertions
1	T	Person or institution to whom an action or omission that requires accountability can be attributed	Subject of responsibility	Persons or institutions
2	U	The person or institution affected by an action or omission of T, for example, harmed by it	Addressee of responsibility	Persons or institutions
3	V	Person or institution authorized to impose sanctions on T	Authority of responsibility	Persons or institutions
4	W	Action or omission that can be attributed to T and is considered to require accountability	Object of responsibility	(Results of) actions or omissions
5	X	T’s portion of responsibility for W	Extent of responsibility	Specifications like “fully” or “partially” or percentage
6	Y	Time interval within which the conditions for the attributability of W to Tare met	Time frame	Time intervals
7	Z	Set of unwritten or codified rules that define the requirement of the accountability of W to T	Normative standard	Rules, regulations, prohibitions, permissions, or systems of those norms

Responsibility as a relational structure can be represented schematically as follows [29, 30]: Subject T is responsible to addressee U and authority V for object W, to an extent X, with regard to a time frame Y, *because of* certain normative standards Z.

The normative standard thus constitutes the moral reference point that enables responsibility to be assigned and interpreted. Yet, to accept such an assignment, a shared normative standard between the responsible subject, the addressees, and relevant authorities is necessary. Otherwise, moral responsibility becomes ambiguous or collapses into purely legal or procedural forms of attribution.

Four preconditions are essential for the assignment of responsibility—voluntariness, level of information, self-determination and accountability, and availability of alternative options [30]. While the normative standard provides an ethical orientation for evaluating whether responsibility can be assigned to an action, the preconditions are necessary for presuming free and intentional action.

Provided that moral diversity is assumed in societies, where no uniform moral framework can be presupposed, two questions emerge:

1. How can a normative standard of responsibility be conceptualized under conditions of moral diversity?
2. What challenges does moral diversity—and the lack of a unified moral standard—pose for a justifiable implementation and use of AI-CDSS?

## Moral diversity and AI-CDSS

### Moral diversity as an ethical challenge

Moral diversity is here foremost an empirical observation grounded in the coexistence of different societies and cultures. This is relevant in healthcare contexts when normative frameworks lead to conflicting assessments of medical interventions, with individuals drawing on a wide variety of moral justifications. This can be illustrated, for instance, by cases in which medical technologies may be rejected as an unacceptable interference with nature or endorsed as a moral obligation to preserve life.

These different moral justifications frequently generate disagreement and moral conflict when incompatible justificatory systems collide [36, 37]. Furthermore, these forms of reasoning—rooted in religion, tradition, emotion, intuition, or reflection—are often incommensurable [36], posing significant challenges for ethical theory and practical decision-making in healthcare.

This diversity raises the question of (ethical) relativism. Descriptive relativism refers to the empirical variety of moral norms, whereas *normative relativism* claims that moral principles are valid only relative to particular individuals and/or groups [37, 38]. Ethical relativism, in this stronger sense, challenges the idea of universal justification and, by extension, the coherence of moral reasoning itself. This creates difficulties for the justification of clinical decisions when different stakeholders operate with incompatible normative standards, a situation that becomes even more pronounced in digitally mediated healthcare environments. This plurality of moral frameworks has also been discussed in the context of global information ethics, where ethical pluralism is considered a structural condition of contemporary digital contexts [39]. Nevertheless, factual diversity does not necessarily entail radical or strong relativism, which would remove all limits to ethical judgment. The challenge is particularly relevant in healthcare, where decisions must still be made despite the absence of a fully shared moral framework. Because the coexistence of multiple moral frameworks may still permit a weak universalism grounded in minimal commonalities—such as a Rawls’s *overlapping consensus* [40], Nussbaum’s [41] basic list of human capabilities, or Beauchamp and Childress [42] proposed mid-level principles of autonomy beneficence, non-maleficence, and justice. While such minimal standards cannot dissolve all theoretical incompatibilities [36], they indicate that complete arbitrariness of norms is untenable.

This underdetermination leads to challenges for decision-making, especially in the context of medical and nursing practice, where different normative assumptions must be reconciled, involving patients, healthcare professionals, and increasingly also AI-based systems.

Therefore, within a *relational concept of responsibility*, moral diversity is a constant challenge because responsibility presupposes shared normative standards as its evaluative basis. If moral relativism were radicalized, no such common ground could exist, and responsibility would be reduced into merely legal (liability) or social (accountability) categories [43].

Hence, in a society that demands moral responsibility, normative relativism is logically inconceivable in the context of responsibility attribution and acceptance. All must share the normative standard as minimal as it may be. A withdrawal from a shared normative standard leads only to incoherent attribution of responsibility.

The following subsections highlight these challenges of normative standards within the context of AI-CDSS, focusing on how moral diversity materializes in algorithmic design, professional–patient relations, and patient perspectives, thereby directly affecting the attribution of responsibility.

### Challenges of moral diversity in AI-CDSS

#### Bias and moral diversity

One of the key challenges of moral diversity and AI-CDSS is biased algorithms. They embody how moral diversity may intersect with technological design. The term itself suggests that algorithms are influenced in a previously (mostly) unintended direction or that a deviation from a predefined norm-set can be detected, be it ethical or statistical [11, 44]. From a relational responsibility perspective, bias in AI systems is not merely a technical deviation, but an indication of how normative assumptions are embedded within socio-technical decision structures. Rajkomar et al. [45] differentiate four areas in which a bias can enter an AI system: design, data, interaction with the medical user and interaction with the affected person. These areas illustrate that responsibility for bias cannot be in a single location, but is distributed across design choices, data generation, clinical interaction, and patient engagement.

At the same time, these are not purely technological problems: morally biased algorithms can also be considered a “social problem”, which requires more than a simplistic solution approach [46]. Of particular interest here is a moral “inclination” which can lead to biased outcome or result from biased input. While an explicit moral implication or decision comes to mind first, other data or design decisions can lead to moral implications or decisions [11, 47]. This implies that addressing bias requires a relational understanding of responsibility, in which epistemic and normative contributions are co-produced across technical and social domains.

Machine learning CDSS are based on data and are therefore also susceptible to bias. For example, a basic distinction can be made between quality and quantity aspects. Thus, in terms of quality, the comparability of input data must be considered, which can lead to bias due to missing, partly existing or incorrect references. Mehrabi et al. [48] present a striking complexity of various biases due to data selection and layout, such as measurement bias, transmitted variable bias, or sampling bias, demonstrating how procedural variations can induce significant distortions with moral implications.

For example, the data may be subject to a moral or anthropological bias such as gender-discriminatory and racist principles that subsequently exclude certain groups as data providers [49], explicitly or implicitly [44]. Alternatively, erroneous calculations, when coinciding with moral attitudes, can cause statistical and moral bias and thus lead to skewed decisions [47, 50].

Similarly, biases may arise during annotation or feedback phases, or through latent effects that manifest with delay [51]. Design choices—like the selection of parameters, computational models or promoting fairness rules—carry ethical commitments [45, 49]. From this perspective, algorithmic design decisions are not neutral but constitute sites of distributed normative influence within AI-CDSS, primarily located in the interaction between developers, healthcare professionals, and patients. Implementing algorithms therefore requires explicit negotiation of which moral conception should guide the system. Following Danks and London [11], one might even speak of a *consciously chosen moral bias*, as “diverse societies exhibit significant variation in both immediate and higher-order relevant values” (p. 4695). The persistent challenge lies in understanding bias not as an isolated error, but as a structural aspect of AI-CDSS. In this context, embedded moral standards may diverge from those of patients and healthcare professionals, thereby complicating and potentially obscuring the attribution of responsibility in clinical decision-making.

#### Professional–patient relationship and moral diversity

**Fig. 1:**
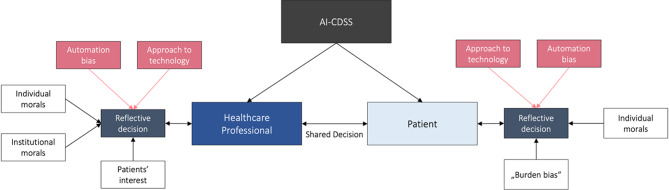
Influences in a professional-patient-relationships towards AI-CDSS in a shared decision process (own illustration)

Moral diversity also manifests in the professional–patient relationship, which can conclude in shared decision making (SDM) and its various forms [52]. This relationship is inherently complex, most times formed by different moral attitudes and expectations (see Fig. 1). In this setting responsibility is primarily distributed between the healthcare professional and the patient within a dyadic decision-making structure. This distribution is relatively stable, as both actors are directly involved in the process.

Challenges apply particularly to the quality of the relationship, such as autonomy, patient-centeredness, communication skills, patient participation, or trust to openly present values and interests [53, 54]. This requires healthcare professionals to recognize and accept patients’ values and interests as well as their role as partners or coaches [53] and to empower patients in their decision-making roles. Within this configuration, responsibility remains largely interpersonal, even if asymmetrically distributed between healthcare professional and patients. This also does not exclude the possibility that patients may want to avoid, to delay or that there are cultural variants in these decisions as Fisher et al. [55] illustrate. Balancing these aspects still seems to be precarious considering past paternalistic role distributions. Yet, these challenges indicate, that “implementing SDM with a ‘one-size fits all’ approach without considering specific contextual factors will not necessarily achieve this goal” [55].

The introduction of AI-CDSS complicates this dynamic of decision-making, as it challenges the moral understanding of both healthcare professionals and patients [56]. AI systems may function here as potential stakeholders in the decision-making process, as they can shape the moral and practical framing of choices. From a relational responsibility perspective, AI systems can be understood as a third quasi-actor in the decision-making constellation. They challenge epistemic authority and complicate the attribution of responsibility across human and non-human contributors. As mentioned, hidden biases within AI systems may conflict with healthcare professionals’ or patients’ values. Since biases or values are not always transparent, they can be unintentional and/or remain hidden even from the developers [48, 49]. Additionally, attitudinal ambivalence may further deepen the problem: patients may simultaneously value technological efficiency and fear dehumanization or loss of control, just as professionals demand an improvement of AI literacy [57, 58].

The interaction between a biased algorithm and an attitudinal bias of the user can create further challenges, for instance when outcomes are uncritically accepted, merely because they are falsely assumed to be objective or neutral technology [47]. An unreflective acceptance can also be caused by an automation bias, which involves the uncritical acceptance of outcomes regardless of their actual reliability [59–61]. This may reduce the level of critical engagement in clinical decision-making and shift how responsibility is practically taken up in the professional–patient interaction. Here, various circumstances such as time pressure, workload, and personal stress can hinder critical reflection on outcomes. However, this lack of reflection might intensify moral values by reinforcing them through algorithms. This dynamic risks marginalizing patient values and creating the illusion of objectivity, thereby eroding genuine moral dialogue.

A normative justification for addressing plurality can be found in principlist approaches. While Beauchamp and Childress’s principlism is often regarded as a practicable framework for addressing moral diversity in clinical decision-making, it relies on the contextual weighting and balancing of ethical principles within shared deliberative practices [42]. However, as Gordon [62] emphasizes, under conditions of technology this balancing becomes increasingly complex due to additional algorithmic and data-driven normative influences, which challenge the stability and transparency of such deliberative processes.

Ultimately, moral diversity remains both a site of tension and a structuring condition for the distribution and reconfiguration of responsibility in AI-CDSS, even when it leads to persistent disagreement about responsibility attribution.

#### Patient perspective and moral diversity

Ultimately, patients as recipients of AI-CDSS encounter unique challenges, which are related to the patients’ preconditions and moral preferences [55]. Their engagement with AI-CDSS is often unprepared and shaped by misconceptions. This already affects how responsibility is perceived, as patients may either overestimate or underestimate the role of the system in decision-making. The very term *artificial intelligence* can evoke various concepts like popular ideas of a cognitive quasi-omnipotence or fears of losing humanity and autonomy [63, 64]. Such perceptions complicate the communication with patients, given they might only be partially aware of the general characteristics and challenges of dealing with AI. Yet this does not limit their values or their personal interests.

But, it highlights the requirements for informed consent, questioning what and how such explanations should be provided. This includes clarifying their moral and normative implications, as well as the potential absence of alternative normative frameworks within the algorithm [65, 66]. Here it becomes less clear who is responsible for ensuring that patients understand not only the medical risks, but also the role and limitations of the AI system.

Given that patients enter an asymmetric dependency relationship with healthcare professionals as information mediators, the competence of professionals becomes critical. In such constellations, responsibility for decision outcomes can be distributed between clinical expertise, system recommendations, and patient trust. Unawareness of moral bias therefore may result in one-sided communication and undermine patient autonomy to decide freely and with full information. Questions, for instance, on reasoning or safety of the system might be left unanswered [66]. Insofar, questions of responsibility already arise with regard to the preconditions of responsibility [30], but also in the interpretation and communication between healthcare professionals and patients.

Finally, patients might also trust AI’s outcomes or recommendations excessively without reflection, especially within challenging situations that create burden and stress [55]. One could almost speak of a “burden bias” in analogy to automation bias [59] (see Fig. 1). In this case, responsibility may gradually shift away from active patient engagement toward a more passive reliance on system-generated recommendations. It is not far-fetched to imagine that outcomes of AI-CDSS are blindly trusted, without bringing forth one’s own needs, interests, or values, and delegating moral agency to technology. This trust might be grounded in an optimism about AI and technology or in an interpersonal relationship with healthcare professionals [65–68]. Nevertheless, it illustrates how moral diversity, unacknowledged or unmanaged, can collapse into moral passivity, where responsibility becomes diffuse and less clearly attributable among patients, healthcare professionals, and the AI-CDSS system.

Taken together, these aspects show that responsibility in AI-CDSS cannot be assigned straightforwardly to single actors, but is instead shaped through the interaction between technology, healthcare practice, and differing moral perspectives, making clear attribution difficult.

## Moral diversity and the normative standard

**Fig. 2:**
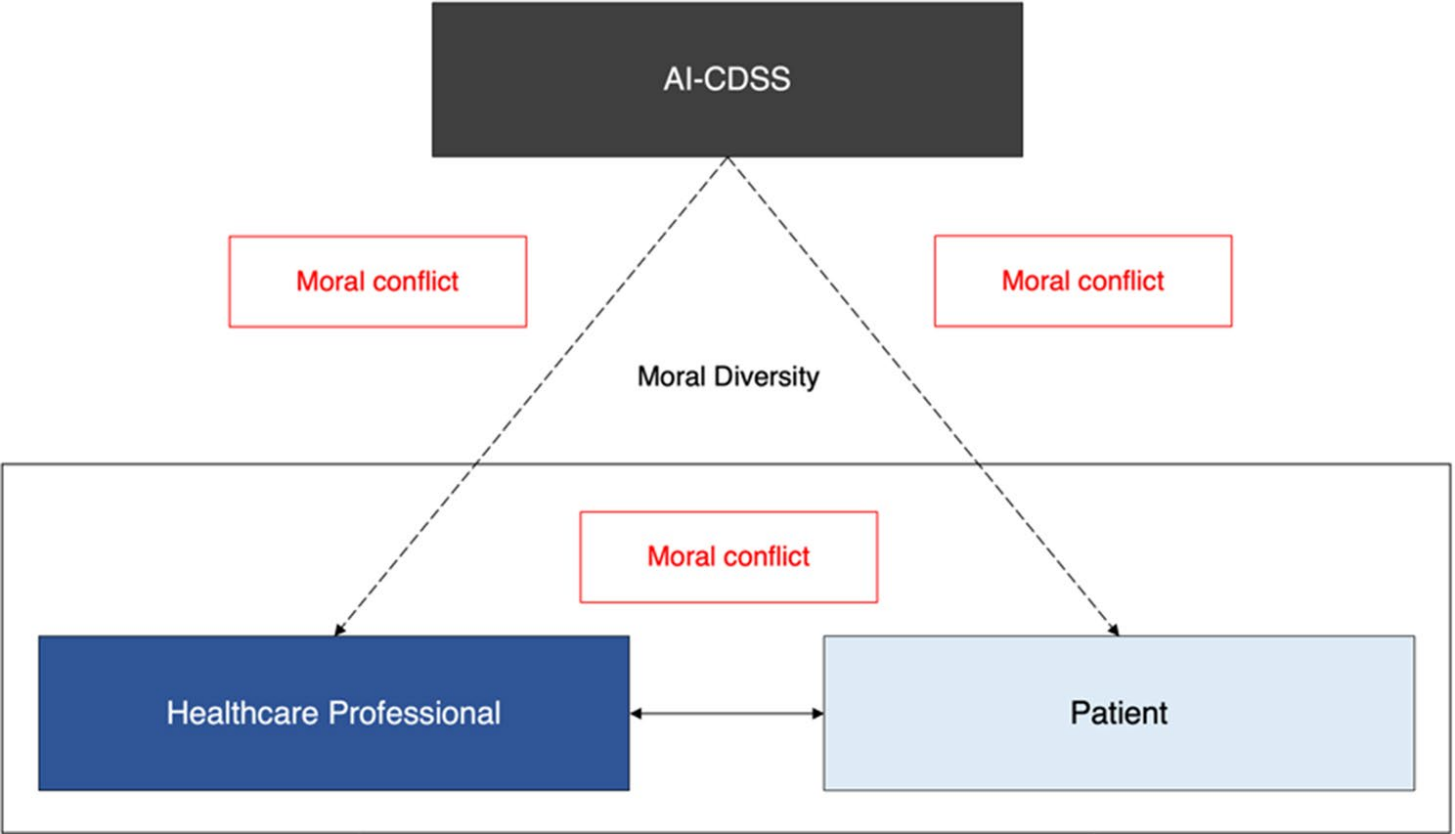
Moral conflicts within the triangle of healthcare professional, patient, and AI-CDSS (own illustration)

Addressing responsibility in AI-CDSS from the perspective of healthcare professionals and patients reveals moral diversity as a fundamental aspect of clinical decision-making among relevant stakeholders—though these can also include developers and healthcare institutions. Yet recognizing this moral diversity intensifies already complex deliberative processes and increases both human-human and human-machine interaction, heightening the potential for moral conflict among healthcare professionals, patients, and AI-CDSS as a quasi-actor AI-CDSS (see Fig. 2).

Comparing the three different relations between healthcare professionals, patient, and AI-CDSS this shows that AI-CDSS cannot be conceived as a genuine moral agent but rather as a *quasi-actor* operating on a non-reflective level. While it can be considered whether technology can be a full moral actor, at least right now it is not [69]. Current systems lack the capacity for moral reflection or intentionality. Nonetheless, patients and healthcare professionals may ascribe quasi-moral agency to AI-CDSS, making it appear as a partner within SDM processes. This aligns with the observation of Longin et al. [70] that assisted systems, especially when they act with AI methods, are less blamed for negative effects. This might be attributed to higher expectations placed on humans than on machines [70, 71]. Yet algorithms are not reflective and self-adjusting moral actors. They lack moral self-awareness and do not contemplate patients’ or healthcare professionals’ needs and interests [56, 72].

Thus, the technology cannot be a subject of responsibility; it remains with humans as moral actors and is formed through socio-technical interaction.

Focusing on human interaction, one might consider the method of SDM as a solution to the challenge of moral diversity. But the integration of AI-CDSS changes the conditions under which SDM takes place. This is relevant given the epistemic and behavioral impact on human cognition and judgment. Unlike traditional rule-based systems, AI-CDSS rely on data-driven and probabilistic models that are not fully transparent to users, thereby affecting the epistemic basis of clinical decision-making. This is relevant in the context of moral diversity, as the normative assumptions embedded in data-driven systems are often implicit rather than explicitly defined, making them harder to identify and evaluate. As seen in Sect. 3.2, AI-CDSS might alter deliberative constellations in ways that are also significant through mechanisms such as automation bias, technological overreliance, and the internalization of algorithmic bias [61]. Thus, moral diversity and responsibility need to be reconsidered in light of a technological integration. Two questions arise: (a) How should the plural moral landscape be addressed in relation to responsibility in AI-CDSS? (b) On which normative standards, which acknowledge or incorporate diversity, can an AI-CDSS be based?

The first problem concerns the pragmatic challenges of translating heterogeneous moral norms into a single operational clinical framework. Two aspects seem particularly prominent here: While AI-CDSS can operate with individual personal data and generate individualized outcomes, they provide limited access to patients’ personal interests and values [56, 73]. It is unclear whether engineering and logical constraints even allow an adaptation of interests and values given the different conceptualizations of implementing ethics in AI systems [18, 74]. Moreover, this raises a further question regarding SDM, which aims at agreement between patient and healthcare professional and whether it can accommodate value pluralism. While SDM should ideally lead to agreement or consensus [75], moral conflicts might also not be solved to the satisfaction of all stakeholders. This prompts normative question: should the patient’s interests and values be prioritized over those of the healthcare professionals? Further, if individualized assessment of patients’ interests and values were possible in AI-use, wouldn’t that result in situational ethics, which would not allow for comparability of cases, thereby lacking evaluability or verifiability? Moral diversity thus reveals a potential limit between aligning human and machine decision-making in healthcare contexts.

The second question concerns the normative foundation of responsibility attribution. As seen, the relational concept of responsibility presupposes a shared normative standard. Given the challenges of moral diversity, a common ground might only be understood as a *minimally shared normative standard* [76].

Establishing a common standard poses challenges for negotiation, stakeholder involvement, and technical implementation. It may constrain individual interests and values depending on whether consensus, compromise, or a pragmatic “muddling through” solution [77] is pursued. Regardless of the interpretation of moral diversity as adaptive and negotiable [78] or as having potential for universalizable norms [42], continuous stakeholder engagement and iterative revision are indispensable. Such standards, necessarily provisional, require ongoing critical monitoring to maintain normative validity.

However, the establishment of common moral standards does not guarantee their practical realization. As shown in Sect. Moral Diversity and AI-CDSS, various algorithmic biases, human–technology asymmetries, and deficits in moral and technical competence may undermine responsible practice. But, these refer primarily to preconditions that enable responsibility (see Sect. 2) rather than responsibility itself. Therefore, these preconditions must also be addressed across moral, technological, interpersonal, and epistemic dimensions [20].

Accordingly, the design of morally responsive technologies requires early and sustained ethical engagement of developers and users. At the same time, this means that cultivating awareness of moral pluralism and the context-dependence of communication is essential [10, 79].

The examples of human-machine and human-human interactions show the amplifying potential of AI-CDSS for generating conflict. AI-CDSS does not change the locus of responsibility, but it alters how responsibility is distributed, perceived, and enacted in clinical decision-making. Especially regarding moral diversity, patients and healthcare professionals require structured education in algorithmic literacy to be able to act responsibly. This includes a basic understanding of the technology and its challenges, but also less obvious skills and pitfalls, such as recognizing ill-considered assumptions and avoiding unreflective adoption of results [22]. Such competence underpins the sustained cooperation between patients and healthcare professionals and the cultivation of a shared ethos of responsibility.

## Conclusion

The analysis of moral diversity reveals complex challenges arising between technology, human moral reasoning, and normative standards within AI-CDSS. Understanding these interconnections is essential for developing a shared foundation for responsible decision-making.

From this, the analysis links *moral diversity* to a *relational concept of responsibility* and characterizes AI-CDSS as a quasi-actor within ethical decision structures. It further contributes insights into how moral pluralism reshapes the attribution of responsibility, thereby situating these reflections within existing debates on responsibility in AI-based healthcare contexts. The findings show that responsibility in AI-CDSS is closely connected to the presence of plural normative standards that shape professional–patient interactions and complicate processes of responsibility attribution.

It can therefore be argued that moral integration requires the establishment of *minimally shared normative standards* as the basis for ethically responsible human-machine interaction.

Such standards enable a practicable yet reflexive distribution of responsibility across all interacting stakeholders. From an applied perspective, this necessitates (1) continuous ethical awareness and education among healthcare professionals and patients, and (2) sustained interdisciplinary discourse extending from the early phases of system design to deployment.

Addressing the challenges posed by moral diversity requires a collective effort to remain attentive to how technology shapes moral reasoning and professional judgment, and to raise awareness of its implications for the development and use of healthcare technologies.

This analysis emphasizes the need for responsible AI systems in healthcare to acknowledge moral diversity as a structural condition of responsibility.

## Data Availability

No datasets were generated or analysed during the current study.
